# Raw Data as the Verification Layer: Why AI-Driven Materials Discovery Needs Experimental Infrastructure

**DOI:** 10.1021/acsomega.6c04971

**Published:** 2026-06-24

**Authors:** Nik Reeves-McLaren

**Affiliations:** School of Chemical, Materials and Biological Engineering, University of Sheffield, Sir Robert Hadfield Building, Mappin Street, Sheffield S1 3JD, United Kingdom

## Abstract

Artificial intelligence models for materials discovery are only as reliable as the data on which they are trained, yet systematic audits across the chemical sciences reveal quantifiable error rates in published experimental and computational data that often exceed the predictive accuracy of these models. Domain experts distinguish real from AI-generated characterization data at accuracy levels indistinguishable from random chance. This Viewpoint argues that the established culture of rigorous data collection, calibration, and metadata documentation that is standard at large experimental facilities represents a model for data integrity that the wider community must now adopt. Protecting and investing in these facilities, and in the expert staff who operate them, is not merely a matter of maintaining experimental capability; it is essential to ensuring that the data driving AI-enabled discovery can be trusted.

## Graphical abstract

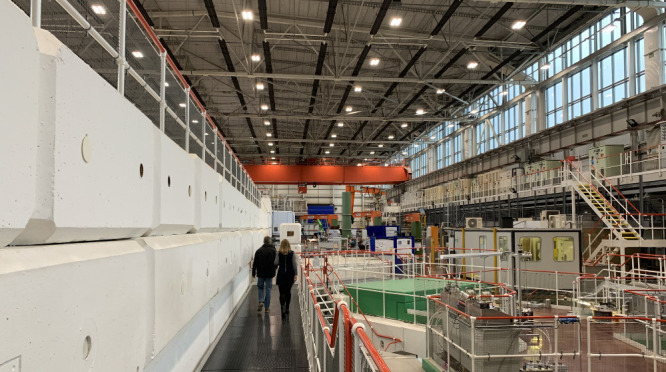

## The Data Quality Problem

It is clear that the future of material discovery will be hugely accelerated by the rise of artificial intelligence. Already, machine learning models have been applied to predict stable inorganic compounds at unprecedented scale,^1^ to design metal–organic frameworks for carbon capture,^2^ and to identify candidate solid-state electrolytes for next-generation batteries.^3^ These models learn structure–property relationships from the vast body of published experimental and computational data and use them to propose candidate materials faster than any experimental program could screen them.^4^

The challenge is that this body of data is considerably less reliable than commonly assumed. The problems are not confined to one technique or subdiscipline; they are structural and span the way many scientists conduct their work.

### We Measure Badly

Systematic audits of routine characterization reveal error rates that would be unacceptable in most engineering disciplines. An automated analysis of over 3,000 Supporting Information files in *Organic Letters* found only 40% compliance with journal guidelines for accurate mass measurements, with approximately 30% of values containing outright errors.^5^ A blind interlaboratory study of 18 international elemental analysis service providers found that roughly one in six carbon determinations failed the ±0.4% threshold required by most chemistry journals.^6^ In X-ray photoelectron spectroscopy, a review of over 1,300 publications found that more than 40% of fitted spectra contained errors serious enough to compromise the reported conclusions, while 85% of papers did not even specify the analytical software used.^7^ These are the techniques we rely on to confirm what we have made and whether it is what we think it is.

### We Analyze Inconsistently

Even when raw experimental data are of high quality, the derived quantities that populate machine learning training sets can vary dramatically depending on who processes them and how. When 61 laboratories were asked to calculate BET surface areas from 18 identical, premeasured adsorption isotherms, round-robin variation coefficients for well-studied materials such as HKUST-1 and ZIF-8 were 7–14%; for NU-1104, the highest reported surface area exceeded the lowest by more than a factor of 5.^8^ On the computational side, a pairwise comparison of three major high-throughput density functional theory databases (AFLOW, Materials Project, and OQMD) found median relative absolute differences of 6% in formation energy, 9% in band gap, and 8% in total magnetization across records derived from identical input crystal structures. Up to 7% of records disagreed on whether a material was metallic, and up to 15% on whether it was magnetic.^9^ This matters: BET surface area and DFT formation energy are among the most commonly used descriptors and target properties in materials machine learning. The spread introduced by inconsistent human analysis or computational parametrization is, in many cases, larger than the predictive accuracy claimed by the models trained on those values.

### We Do Not Check

The scientific record rests on an assumption of independent verification that, in practice, rarely occurs. Eighty-nine percent of reported metal–organic framework materials had never been independently resynthesized within 5–11 years of publication.^10^ *Organic Syntheses*, one of the very few journals that requires mandatory independent replication, rejected 7.5% of submissions between 2010 and 2016 for irreproducibility, a figure that likely represents a lower bound for the wider literature where no such checks exist.^11^ In crystallography, approximately 800 fabricated papers from a single paper mill passed peer review across multiple major publishers.^12,13^ My own automated bond valence sum screening of 840 rock salt structures in the Inorganic Crystal Structure Database, the simplest crystal structure we have, found that only 50.8% validated, with failure modes ranging from systematic parameter inadequacies to multiphase refinement contamination.^14^ If we struggle to get rock salts right, what issues will future studies find with the more complex structures that make up the bulk of functional materials? An analysis^100^ of 1,292 chemistry retractions between 2001 and 2021 found that 58.5% were attributable to misconduct, with crystallography disproportionately affected by data fabrication specifically.^15^ The majority of these retracted papers had been cited, and many remain in the databases that train AI models. Data from such work must also be systematically identified and removed from databases, but poor-quality data must also be pruned. Automated screening tools are emerging for crystallographic databases,^14^ but equivalent efforts are needed across characterization techniques and data types.

### We Can No Longer Detect the Fakes

Generative AI has made the fabrication of plausible characterization data trivially easy and its detection near-impossible with current methods. Researchers have demonstrated that convincing fake atomic force microscopy, scanning transmission electron microscopy, and transmission electron microscopy images can be produced in under an hour using commercially available tools. When 250 scientists were presented with real and AI-generated microscopy images in a blind survey, expert accuracy in distinguishing them was 40–51%: indistinguishable from random chance.^15^ My own tests showed that reusable Python code to manipulate diffraction data or smooth long-term battery cycling curves could be generated using large language models, such as Google’s Gemini, without ethical challenge in minutes.^13^

If the data underpinning AI models contain quantifiable, systematic errors across analytical, synthetic, structural, and computational chemistry, and if domain experts cannot reliably distinguish authentic from fabricated characterization data, then the integrity of training data sets will be a critical challenge for AI-driven materials discovery. This is not a future risk but a present reality.

## Raw Data as the Verification Layer

The question that emerges is not simply practical but epistemological: when published data contain systematic errors, when those errors propagate unchecked into AI training corpora, and when fabricated data are indistinguishable from authentic results, what constitutes a reliable scientific result in the chemical sciences—or any STEM field? The traditional answer, expert judgment informed by experience, has been shown to be inadequate. We need characterization data that are inherently verifiable, where the act of measurement itself leaves traces that are difficult to forge. Raw instrument files from well-maintained, well-calibrated instruments can offer this, and large-facility facilities offer a particularly strong example.

Consider a powder X-ray diffraction pattern. The processed output that many researchers will aim for is a two-column text file of angle and intensity that can be fabricated or manipulated using generative AI in minutes, often without question or challenge.^13^ However, the raw instrument file generated by a modern diffractometer or a synchrotron beamline is a fundamentally different object. At facilities such as Diamond Light Source or ISIS Neutron and Muon Source, raw data are stored in the NeXus/HDF5 format, an international standard adopted across neutron, X-ray, and muon science.^16^ A NeXus file is not simply a data container; it is a structured hierarchy of metadata encoding the radiation source type and energy, monochromator wavelength, detector model and calibration state, collection timestamps, sample environment parameters, and instrument geometry.^16,17^ These fields are not independent. Beam energy, detector response, and monochromator settings must be mutually consistent for a specific beamline configuration at a specific date. Fabricating a convincing raw file would require not just knowledge of the data format but detailed, internally consistent knowledge of the instrument state at the time the data were purportedly collected, information that is rarely if ever publicly documented at the level of specificity required. These metadata do not appear by accident. It is the product of decades of institutional investment in calibration protocols, instrument characterization, and data management practice, maintained by specialist staff whose expertise is too rarely visible in the publications that rely on it. The raw file is the artifact; the culture of rigorous data stewardship at these facilities is a safeguard.

Laboratory diffractometers generate analogous, albeit less complex, raw files. Formats such as Bruker’s .raw and PANalytical’s .xrdml embed scan parameters, tube voltage and current, detector type, goniometer configuration, and collection timestamps. These metadata are recorded automatically by the instrument and are not routinely accessible for manual editing. The principle applies across characterization techniques: the richer the embedded metadata in a raw instrument file, the harder that file is to fabricate convincingly.

This observation has practical consequences for the data integrity policy. The Minimal Arrangement of Instrument Files (MAIF) framework, proposed by Davydiuk et al. in the context of nanomaterials microscopy, provides a simple, standardized directory structure for depositing raw data alongside publications: each manuscript has its own folder, each figure has a subfolder containing the primary instrument files, and nonfigure data are stored separately.^15^ With a colleague, I previously proposed extending this principle through a modular data integrity checklist with a core module applicable to all materials science submissions and technique-specific modules (for X-ray diffraction, electrochemistry, and other methods) that define what raw data and metadata should be deposited and what validation checks should be applied. The checklist includes requirements for structured raw data deposition following MAIF or similar principles, with the explicit recommendation that journals move from encouraging to mandating raw data files as a publication criterion.^13^

The argument extends beyond the individual publication integrity. Large-facility experimental facilities—synchrotrons, neutron and muon sources—generate data of a quality and provenance that cannot be replicated computationally. The relationship between experimental infrastructure and AI is not one of competition; it is one of dependence. AlphaFold, perhaps the most celebrated example of AI in the physical sciences, was trained on the Protein Data Bank (PDB). Approximately 85% of the crystallographic structures in the PDB were determined using synchrotron radiation, a proportion that has exceeded 90% for new depositions in recent years.^17,18^ Without synchrotron beamlines, and the excellent staff to support and run them, there is no training data, and by extension no AlphaFold. The same logic applies to materials science: the battery sector’s ambitions for digital twins and thermal runaway prediction depend on *operando* characterization data, collected principally at synchrotron and neutron facilities. These data tell the physical story of what actually happens inside the cells during operation. You cannot model what you do not measure.

## Experimental Infrastructure as an Integrity Safeguard

The argument presented here reframes large-facility experimental infrastructure in terms that are rarely articulated in funding discussions. Synchrotrons, neutron sources, and muon facilities are typically justified on the basis of the science they enable: the unique experiments, the high-resolution data, and the techniques unavailable elsewhere. These justifications are sound, and they have been well rehearsed. What is less commonly recognized is the role these facilities play as guarantors of data integrity and good practice at a time when the trustworthiness of scientific data is under pressure from multiple directions simultaneously.

This reframing is not an abstraction. In the UK, a recent open letter signed by over 400 researchers across more than 60 institutions highlighted the dependence of the broader research community on the large-scale national facilities operated by the Science and Technology Facilities Council (STFC), including Diamond Light Source, ISIS Neutron and Muon Source, and the Central Laser Facility. The letter noted that at Diamond, over 90% of UKRI-funded research activity is supported not by STFC but by other research councils, including EPSRC, BBSRC, and MRC.^19^ In their response, the UKRI Chief Executive and STFC Executive Chair acknowledged these facilities as critical national assets underpinning a broad and interdisciplinary research base, while confirming that STFC faces cost pressures that are structurally unique among UKRI councils and that some pre-existing programs will need to be reduced in scope and budget.^20^ The response was committed to a whole-system perspective and cross-council assessment of facility dependence.

The data integrity argument developed in this Viewpoint adds a dimension to that discussion that has not, to my knowledge, been made explicitly. The AI strategy of every major research funding nation rests on the assumption that reliable training data exist. In materials science, the most reliable data come disproportionately from large facilities, not simply because of the instruments themselves but because of the institutional culture surrounding them: the calibration regimes, the standardized data formats, the sample environment documentation, and the expert staff who maintain these practices as a matter of routine rather than exception. To prioritize AI-driven materials discovery while reducing the capacity of the experimental infrastructure that generates world-leading science *and* trustworthy training data would be to undermine the foundations of the very program being promoted. This is not a tension between competing priorities; it is an interdependence that research policy must recognize explicitly. This argument is not confined to the UK.

The practical implications are straightforward. First, funding bodies should require that raw instrument files from publicly funded experiments be deposited in structured, openly accessible formats as a condition for grant. Repositories such as Zenodo, the Open Science Framework, and Figshare already support this; what is lacking is a consistent mandate rather than an aspiration. This must also include metadata to make the files useful and accessible.

Second, facility data policies should be designed with downstream AI use in mind. If synchrotron and neutron data are to serve as training sets and validation benchmarks, they must be findable, accessible, interoperable, and reusable in practice, not just in principle.^21^

Finally, the community of practice around data integrity needs to extend beyond individual research groups to encompass professional societies, journal editors, and funding panels. The modular checklist approach I have previously proposed with colleagues,^13^ the MAIF framework for raw data deposition,^15^ and automated database-quality tools such as Eir^14^ are starting points, not end points. They require testing, refinement, and adoption at a scale that no single group can achieve alone. Critically, this also means investing in the people who make data integrity possible: the beamline scientists, instrument specialists, and data managers whose work underpins every data set but who are often the first to face cuts or not be replaced when budgets are constrained.

## Closing Remarks

The argument made here is simple, but its implications are profound. AI-driven material discovery depends on training data of known provenance and verified quality. The published scientific record, as we have shown, falls short of this standard across analytical, synthetic, structural, and computational chemistry with quantifiable error rates that often exceed the predictive accuracy claimed by the machine learning models trained on that data. Domain experts cannot reliably distinguish authentic from fabricated characterization data. Existing verification practices, peer review, independent replication, and database curation operate at a scale and rigor that are inadequate for the volume and pace of modern data generation.

The culture of rigorous data stewardship at large experimental facilities, and the metadata-rich raw files it produces, offers a model for how the wider community might respond. None of this is an argument against AI in materials science—its potential is real, and early progress is genuine. It is an argument for building on solid ground. The community has the tools, or the beginnings of them: structured raw data deposition through frameworks like MAIF,^15^ modular integrity checklists for common characterization techniques,^13^ automated database-quality screening at scale.^14^ What is needed now is the will to adopt them as standard practice rather than as optional extras, and the recognition that the facilities, the staff, and the culture of rigorous experimental practice that together generate our most trustworthy data are not legacy costs to be minimized but essential components of the AI-enabled future we are trying to build.

The foundations must be sound before we add stories.

## References

[1] Merchant A., Batzner S., Schoenholz S. S., Aykol M., Cheon G., Cubuk E. D. (2023). Scaling Deep Learning for Materials Discovery. Nature.

[2] Park H., Yan X., Zhu R., Huerta E. A., Chaudhuri S., Cooper D., Foster I., Tajkhorshid E. (2024). A Generative
Artificial Intelligence Framework Based on a Molecular Diffusion Model
for the Design of Metal-Organic Frameworks for Carbon Capture. Commun. Chem..

[3] Sendek A. D., Cubuk E. D., Antoniuk E. R., Cheon G., Cui Y., Reed E. J. (2019). Machine Learning-Assisted Discovery of Solid Li-Ion
Conducting Materials. Chem. Mater..

[4] Butler K. T., Davies D. W., Cartwright H., Isayev O., Walsh A. (2018). Machine Learning
for Molecular and Materials Science. Nature.

[5] Christmann M. (2025). What I Learned
from Analyzing Accurate Mass Data of 3000 Supporting Information Files. Org. Lett..

[6] Kuveke R. E. H., Barwise L., van Ingen Y., Vashisth K., Roberts N., Chitnis S. S., Dutton J. L., Martin C. D., Melen R. L. (2022). An International
Study Evaluating Elemental Analysis. ACS Cent.
Sci..

[7] Major G. H., Fairley N., Sherwood P. M. A., Linford M. R., Terry J., Fernandez V., Artyushkova K. (2020). Practical
Guide for Curve Fitting
in X-Ray Photoelectron Spectroscopy. J. Vac.
Sci. Technol. A.

[8] Osterrieth J. W. M., Rampersad J., Madden D., Rampal N., Skoric L., Connolly B., Allendorf M. D., Stavila V., Snider J. L., Ameloot R., Marreiros J., Ania C., Azevedo D., Vilarrasa-Garcia E., Santos B. F., Bu X.-H., Chang Z., Bunzen H., Champness N. R., Griffin S. L., Chen B., Lin R.-B., Coasne B., Cohen S., Moreton J. C., Colón Y. J., Chen L., Clowes R., Coudert F.-X., Cui Y., Hou B., D’Alessandro D.
M., Doheny P. W., Dincǎ M., Sun C., Doonan C., Huxley M. T., Evans J. D., Falcaro P., Ricco R., Farha O., Idrees K. B., Islamoglu T., Feng P., Yang H., Forgan R. S., Bara D., Furukawa S., Sanchez E., Gascon J., Telalović S., Ghosh S. K., Mukherjee S., Hill M. R., Sadiq M. M., Horcajada P., Salcedo-Abraira P., Kaneko K., Kukobat R., Kenvin J., Keskin S., Kitagawa S., Otake K., Lively R. P., DeWitt S. J. A., Llewellyn P., Lotsch B. V., Emmerling S. T., Pütz A. M., Martí-Gastaldo C., Padial N. M., García-Martínez J., Linares N., Maspoch D., Suárez del Pino J. A., Moghadam P., Oktavian R., Morris R. E., Wheatley P. S., Navarro J., Petit C., Danaci D., Rosseinsky M. J., Katsoulidis A. P., Schröder M., Han X., Yang S., Serre C., Mouchaham G., Sholl D. S., Thyagarajan R., Siderius D., Snurr R. Q., Goncalves R. B., Telfer S., Lee S. J., Ting V. P., Rowlandson J. L., Uemura T., Iiyuka T., van der Veen M. A., Rega D., Van Speybroeck V., Rogge S. M. J., Lamaire A., Walton K. S., Bingel L. W., Wuttke S., Andreo J., Yaghi O., Zhang B., Yavuz C. T., Nguyen T. S., Zamora F., Montoro C., Zhou H., Kirchon A., Fairen-Jimenez D. (2022). How Reproducible Are Surface Areas Calculated from
the BET Equation?. Adv. Mater..

[9] Hegde V. I., Borg C. K. H., del
Rosario Z., Kim Y., Hutchinson M., Antono E., Ling J., Saxe P., Saal J. E., Meredig B. (2023). Quantifying Uncertainty in High-Throughput Density
Functional Theory: A Comparison of AFLOW, Materials Project, and OQMD. Phys. Rev. Mater..

[10] Agrawal M., Han R., Herath D., Sholl D. S. (2020). Does Repeat Synthesis in Materials
Chemistry Obey a Power Law?. Proc. Natl. Acad.
Sci. U. S. A..

[11] Bergman R. G., Danheiser R. L. (2016). Reproducibility in Chemical Research. Angew. Chem., Int. Ed..

[12] Bimler D. (2022). Better Living
through Coordination Chemistry: A Descriptive Study of a Prolific
Papermill That Combines Crystallography and Medicine. ResearchSquare.

[13] Reeves-McLaren N., Moth-Lund
Christensen S. (2026). Data Integrity in Materials Science
in the Era of AI: Balancing Accelerated Discovery with Responsible
Science and Innovation. J. Mater. Chem. A.

[14] Reeves-McLaren N. (2026). Automated
Bond Valence Sum Analysis for Crystallographic Database Quality Assessment:
A Systematic Study of Rock Salt Oxides, Halides, and Chalcogenides. Dalton Trans..

[100] Sevryugina Y., Jimenez R. (2023). ACS Omega.

[15] Davydiuk N., Krieg E., Gaitzsch J., McCall P. M., Auernhammer G. K., Yang M., Tracy J. B., Bals S., Parak W. J., Kotov N. A., Liz-Marzán L.
M., Fery A., Faria M., Besford Q. A. (2025). The Rising Danger of AI-Generated
Images in Nanomaterials Science and What We Can Do about It. Nat. Nanotechnol..

[16] Konnecke M., Akeroyd F. A., Bernstein H. J., Brewster A. S., Campbell S. I., Clausen B., Cottrell S., Hoffmann J. U., Jemian P. R., Mannicke D., Osborn R., Peterson P. F., Richter T., Suzuki J., Watts B., Wintersberger E., Wuttke J. (2015). The NeXus Data Format. J. Appl.
Crystallogr..

[17] Burley S. K., Berman H. M., Duarte J. M., Feng Z., Flatt J. W., Hudson B. P., Lowe R., Peisach E., Piehl D. W., Rose Y., Sali A., Sekharan M., Shao C., Vallat B., Voigt M., Westbrook J. D., Young J. Y., Zardecki C. (2022). Protein Data Bank: A Comprehensive
Review of 3D Structure Holdings and Worldwide Utilization by Researchers,
Educators, and Students. Biomolecules.

[18] Jiang J., Sweet R. M. (2004). Protein Data Bank
Depositions from Synchrotron Sources. Journal
of Synchrotron Radiation.

[19] McCluskey, A. ; Pitcairn, J. ; Cumby, J. ; Hobday, C. ; Jarvis, A. ; Potter, M. Open Letter Regarding The Importance of STFC Facilities. https://stfc-open-letter.co.uk/letter.html.

[20] Chapman, I. ; Dougherty, M. Response to your letter of support for STFC Multidisciplinary Facilities. https://stfc-open-letter.co.uk/response.pdf.

[21] Wilkinson M. D., Dumontier M., Aalbersberg Ij. J., Appleton G., Axton M., Baak A., Blomberg N., Boiten J.-W., da Silva
Santos L. B., Bourne P. E., Bouwman J., Brookes A. J., Clark T., Crosas M., Dillo I., Dumon O., Edmunds S., Evelo C. T., Finkers R., Gonzalez-Beltran A., Gray A. J. G., Groth P., Goble C., Grethe J. S., Heringa J., ’t Hoen P. A.
C., Hooft R., Kuhn T., Kok R., Kok J., Lusher S. J., Martone M. E., Mons A., Packer A. L., Persson B., Rocca-Serra P., Roos M., van Schaik R., Sansone S.-A., Schultes E., Sengstag T., Slater T., Strawn G., Swertz M. A., Thompson M., van der
Lei J., van Mulligen E., Velterop J., Waagmeester A., Wittenburg P., Wolstencroft K., Zhao J., Mons B. (2016). The FAIR Guiding
Principles for Scientific Data Management and Stewardship. Sci. Data.

